# Concept of defensive medicine and litigation among Sudanese doctors working in obstetrics and gynecology

**DOI:** 10.1186/s12910-016-0095-3

**Published:** 2016-02-09

**Authors:** AbdelAziem A. Ali, Moawia E. Hummeida, Yasir A. M. Elhassan, Wisal O. M.Nabag, Mohammed Ahmed A. Ahmed, Gamal K. Adam

**Affiliations:** Department of Obstetrics and Gynecology, Faculty of Medicine, Kassala University, P.O. Box 496, Kassala, Sudan; School of Medicine, Alneelain University, Khartoum, Sudan; Faculty of Medicine, Kordofan University, ElObeid, Sudan; Faculty of Medicine, Alzaiem Alazhari University, Al-ShaBiyya, Sudan; Faculty of Medicine, Gadarif University, AlGadarif, Sudan

**Keywords:** Defensive, Litigation, Medicine, Obstetrics, Gynaecology, Sudan

## Abstract

**Background:**

Obstetrics and gynaecology always has reputation for being a highly litigious. The field of obstetrics and gynaecology is surrounded by different circumstances that stimulate the doctors to practice defensive medicine.

**Methods:**

This study was directed to assess the extent and the possible effect of defensive medicine phenomenon (in term of knowledge and prevalence) on medical decision making among different grades of obstetric and gynaecologic Sudanese doctors, and to determine any experience of medical litigations with respect to sources and factors associated with it (in term of area of work, characteristics of the area at which the doctors worked, professionalism, hospitals systems…ect).

**Results:**

A total of 117 doctors were approached, their distribution according to job description was as follow: consultants (42.7 %, 50\117) registrars (34.2 %, 40\117) and specialists (23.1 %, 27\117). The majority 89.7 % had the impression that litigation against doctors are increasing and 27.6 % had a direct experience of litigation. In this study less than one half (42.7 %) of the surveyed doctors knew the concept of defensive medicine and 71.8 % reported practicing one or another form of defensive medicine. The different sources of the litigations reported by the doctors included: maternal death (*n* = 15), perinatal death (*n* = 5), other {misdiagnosis, intra-uterine fetal death, uterine perforation, rupture uterus} (*n* = 4), fetal distress (*n* = 3), injury to viscera (*n* = 3) and shoulder dystocia (*n* = 2). In this study the experience of medical litigation was significantly observed among those who worked in area of blame culture (90.6 % Vs 56.5 %, *P* = 0.000). In logistic regression model, there was no significant difference between those who knew the concept of defence medicine and those who didn’t.

**Conclusion:**

There should be strategic plan to reduce the practice of defensive medicine and medical litigation against doctors.

**Electronic supplementary material:**

The online version of this article (doi:10.1186/s12910-016-0095-3) contains supplementary material, which is available to authorized users.

## Background

Defensive medicine is defined as a doctor’s deviation from the usual practice in order to reduce or prevent criticism and\or complaints by patients or their relatives [1, 2]. Some would claim that it is a legitimate phenomenon, while others consider it immoral [3]. In addition to this definition the United States Congress also include the action of ordering tests, procedures and visits, or avoidance of high risk patients or procedures with the primary (but not sole) aim of reducing mal-practice liability as a part of defensive medicine [4]. A genuine difficulty exists when trying to identify and quantify the extent of defensive medicine practices. This is partially because there is a grey area between proper and overly self-protective treatment. It may be difficult to recognize medical actions that are more likely to result in legal action. Obstetricians and Gynaecologists like other health care professional have a legal obligation to adhere to reasonable standards of care while acting in their professional capacity, they always has reputation for being a highly litigious [5]. Their field is surrounded by different circumstances that stimulate them to practice defensive medicine. About 5–7.4 % of physicians in USA faced a malpractice claim annually [6]. Gynaecology alone had the 12th highest average annual proportion of physicians with a claim, with the highest payment rate (38 %) [6]. Obstetrics and general surgery are regarded as high risk specialties [7]. As a result, the rising cost of malpractice insurance in obstetrics and genecology has led to a reality where doctors may refrain from treating high risk patients [7]. Again medical litigation represents a real threat for the doctors and may be a direct cause to leave the profession. Medical law is the aspect of the law which governs the relationship between the healthcare provider and patient [8]. The medical practitioner is bound by certain laws depending on the circumstances of his practice. Law and ethics may overlap since obtaining patient permission is both legally required and the “right thing to do” [8]. The Sudan Medical Council (SMC) standing disciplinary committee investigates any complaint that come to its notice or violation to medical ethics. The SMC has the power to erase doctors from its register or withhold the license of medical, dental or pharmacy institution or facility. Many studies were done worldwide concerning the medical litigation especially against obstetrics and gynaecology however none was carried out in Sudan thus this study was directed to assess the concept of defensive medicine (in term of knowledge and prevalence) and to determine any experience of medical litigations and their sources among different grades of Sudanese doctors working in obstetrics and gynaecology.

## Methods

### Hypothesis

While we did not adopt a formal hypothesis for this study, our working hypothesis/assumption was that defensive medicine affects daily doctor’s clinical judgement and practice.

### Study design and data collection

This study was directed to assess the extent and the possible effect of defensive medicine phenomenon (in term of knowledge and prevalence) on medical decision making (development of tools that can guide physicians to make good decisions in practice) among different grades of obstetric and gynaecologic doctors, and to determine any experience of medical litigations with respect to sources and factors associated with it (in term of area of work, characteristics of the area at which the doctors worked, professionalism, hospitals systems…ect). Using a self administered questionnaire (Additional file 1) and after obtaining informed written consent the data was collected from the different certified OBGYN professionals (Registrars, Specialists and Consultants) working in obstetrics and gynaecology and who attended the 27th congress of obstetrical and gynaecological society of the Sudan held from (20th -23rd February 2015) in Khartoum. The survey included only Sudanese doctors who were practicing obstetrics and gynaecology in Sudan. Visiting Doctors who are practicing in a different context abroad were excluded from the study. We used a questionnaire which was constructed by the authors to consider different forms of defensive medicine and medical litigation in obstetrics and gynaecology. Information sought by the questionnaire included: socio-demographic characteristics (age, grade, gender, area of work, duration of work, health insurance coverage), information on the area of work (blame culture: which defined as no one accepts medical errors as being all right), information on the hospital where the respondent worked (hospital guidelines and protocol, auditing system, committees…ect) daily experience (informed consent, high risk consent, documentation), whether the respondents knew the concept of defensive medicine or not? and questions on different examples of positive and negative defensive medicine (prescription of unnecessary medication, experience unnecessary refer, refuse to manage high risk patient, request for unnecessary investigation, experience unnecessary surgical procedure and avoidance high risk surgical procedure because of fear of criticism or litigation). Other information obtained from the respondents included: is the litigation in OBGYN increasing?, whether the respondents experienced litigations during their daily practice and the source of the litigations (fetal distress, misdiagnosis, injury to the viscera, shoulder dystocia, death…ect). The definition of defensive medicine was framed by the investigators according the definition in the literature [1]; defined as a doctor’s deviation from their usual behaviour or that considered good practice, to reduce or prevent complaints or criticism by patients or their families. This definition was not set out in the questionnaire for the respondents however in the questionnaire we asked the respondents whether they know the defensive medicine or not. Defensive medical practices were further subcategorized into positive and negative practice. When extra tests and procedures are performed primarily to reduce malpractice liability, this is a positive defensive medicine. Negative defensive medicine consists of avoidance of certain patients and procedures, thereby withdrawing medical services, and can deny patients productive care [2]. In the questionnaire we explained the situation of high risk consent which is taken in case of serious / complicated / risky / new - surgeries or procedures; for removing any organ; in high risk patients; for proceeding with a surgery / procedure in spite of any abnormal parameters of the patient. This list is indicative not exhaustive and in case of a dilemma it is always advisable to take this high-risk consent and not a general consent.)

### Statistics

Data were entered into a computer database and SPSS software (SPSS Inc., Chicago, IL, USA, version 16.0) and double checked before analysis. Chi-squire test was used and *P* < 0.05 was considered significant. Univariate and multivariate analyses were performed. Defensive medicine was the dependent variable and other variables were independent factors. Confidence intervals of 95 % were calculated and *P* < 0.05 was considered significant. In case of discrepancy between the results of univariate and multivariate analyses, the later was taken as final.

### Ethics

The study received ethical clearance from the Health Research Board at Ministry of Health, Kassala State, and Sudan Medical Specialization Board (SMSB), Obstetrics and Gynaecology Department, Sudan.

## Results

### Characteristics of the respondents and area of work

A total of 117 doctors were approached, their age ranged from 26 to 73 years. Their distribution according to job description was as follow: consultants (42.7 %, 50\117) registrars (34.2 %, 40\117) and specialists (23.1 %, 27\117), Table 1. Of them 106 (90 %) worked in teaching hospital, 11 (9.4 %) in rural hospitals, again 39 (33.3 %) of the respondents claimed that they worked in blame free culture while 78 (66.7 %) believed the opposite. More than half of the participants were female 60\117 (51.3 %) and the majority (76\117, 65 %) were not covered by health insurance. With regard to duration of experience in obstetrics and gynaecology 14.5 % had an experience of less than 5 years, (41.9 %) were of 5–10 years and 43.6 % were more than 10 years of experience. Again with respect to the area of work the vast majority of the investigated doctors mentioned that their hospitals having guidelines and protocol (58.1 %), auditing system (72.6 %) however only 45.3 % and 39.3 % reported having high risk and ethical committees respectively. Respondents reports on daily experience and practices with regards to documentations/and communications was quite variable: they always (61.5 %), usually (29.1 %) and sometimes (9.4 %) applied informed consent, always (55.6 %) usually (25.6 %) and sometimes (18.8) applied high risk consent and they always (39.7 %), usually (43.6 %) and sometimes (17.1 %) documented their findings and intervention.

**Table 1:** Characteristics of the respondents and the area of work (*n* = 117)

Variable	Frequency	Percentage
Job description		
Consultant	50	42.7 %
Registrar	40	34.2 %
Specialist	27	23.1 %
Duration of work		
< 5 years	17	14.5 %
5–10 years	49	41.9 %
≥ 10 years	51	43.6 %
Distribution per gender		
Female	60	51.3 %
Male	57	48.7 %
Hospital		
Teaching	106	90 %
Rural	11	10 %
Culture of the area of work		
Blame area	39	33.3 %
Blame free area	78	66.7 %
Health insurance coverage for the respondents		
Covered	41	35 %
Not covered	76	65 %

### Medical litigation

The majority 89.7 % (*n* = 105) had the impression that litigation against doctors are increasing and 27.6 % (*n* = 32) had a direct experience of litigation. The different sources of the litigations reported by the doctors included: maternal death (*n* = 15), perinatal death (*n* = 5), other {misdiagnosis, intra-uterine fetal death, uterine perforation, rupture uterus} (*n* = 4), fetal distress (*n* = 3), injury to viscera (*n* = 3) and shoulder dystocia (*n* = 2).

### Defensive medicine

Less than one half (50\117, 42.7 %) of the surveyed doctors knew the concept of defensive medicine and 71.8 % (*n* = 84) reported practicing one or another form of defensive medicine. With further classification of defensive medicine; 48 (41 %) reported practicing positive defensive medicine while 36 (30.8 %) reported practicing negative one. Arranging un-necessary refer was the most common form of defensive medicine practiced by the investigated doctors (*n* = 27, 23.1 %) followed by avoiding high risk procedure (*n* = 24, 20.5 %) and ordering unnecessary investigations (*n* = 14, 12 %). Among our respondents 7 (6 %) prescribed un-necessary medication to avoid litigation and criticism, 6 (5.1 %) refused to manage high risk patient because of fear from litigation and 6 (5.1 %) performed un-necessary surgery (caesarean section) to avoid litigation and criticism.

### Factors associated with medical litigation and defensive medicine

In this study the experience of medical litigation was significantly observed among those who worked in area of blame culture (90.6 % Vs 56.5 %, *P* < 0.001), Table 2 while in logistic regression model the different variables (duration of work, qualification, place of work and area of blame culture) were not associated with the concept of defence medicine, Table 3.

**Table 2:** Factors associated with medical litigation among Sudanese doctors working in obstetrics and gynaecology, 2015

Variables	Experienced litigation (*N* = 32)	Didn’t experience litigation (*N* = 85)	*P*
Area of work, teaching hospital	28 (87.5)	77 (90.6)	0.357
Hospital protocol, yes	16 (56.2)	50 (58.8)	0.454
High risk committee, yes	14 (43.8)	39 (45.9)	0.481
Hospital auditing system, yes	21 (65.6)	64 (75.2)	0.179
Area of blame culture, yes	29 (90.6)	48 (56.5)	0.000
Hospital ethical committee, yes	13 (40.6)	33 (38.8)	0.529
Informed consent, always	19 (59.4)	53 (62.3)	0.917
High risk consent, always	13 (40.6)	51 (60.0)	0.067

Data are shown as number (%) as applicable

**Table 3:** Factors associated with concept of defence medicine among Sudanese doctors working in obstetrics and gynaecology, 2015, using univariate and multivariate analyses

Variable	Univariate analyses			Multivariate analyses		
	OR	95 % CI	*P*-value	OR	95 % CI	*P*-value
Duration of work ≥ 5 year	0.9	0.9–1.0	0.017	0.9	0.9–1.0	0.154
Qualification, consultant	1.9	1.1–3.1	0.009	1.6	0.9–2.8	0.081
Place of work, teaching hospital	0.6	1.0–2.0	0.409	0.4	1.0–1.6	0.217
Area of blame culture	1.3	0.5–2.8	0.509	1.4	0.6–3.3	0.372

*Abbreviations: OR* odds ratio, *CI* confidence interval

## Discussion

To our knowledge this is the first published data on concept of defensive medicine and medical litigation among Sudanese doctors. The majority 89.7 % had the impression that litigation against doctors are increasing and 27.6 % had a direct experience of litigation. In this study less than one half (42.7 %) of the surveyed doctors knew the concept of defensive medicine and 71.8 % reported practicing one or another form of defensive medicine. Worldwide there is a growing awareness of the need for more effective communication among caregivers, patients, and their families [9]. With the increasing rates of negligence, patients are beginning to seek redress and are being enlightened by legal practitioners. Health care practitioners are thus confronted with the problem and risk of being sued. This is believed to have influence various aspects of gynecological and obstetrical practice [10]. Thus the National Heath Care system in Sudan should insures medical staff employees, providing compensation to victims of alleged malpractice, including reasonable court fees. This will allow the health practitioners to exercise their minds individually and jointly to effectively give better service to patients. Less than one half of the investigated doctors in this study practiced defensive medicine. Defensive medicine was reported in 96 % among the USA neurosurgeons and in Europe 94 % of the gastroenterologists reported practicing defensive medicine [11]. The practice of defensive medicine has also spread to Italy where 83 % of the surgeons and anesthetists reported practicing defensive medicine [12, 13]. In Japan 98 % of the gastroenterologists also reported practicing at least one or another form of defensive medicine [14]. This discrepancy might be attributed to the culture of area and people motivation and awareness. In 1991 *Ennis et al*. investigated the members and fellows of the Royal College of Obstetricians and gynecologists and found that the majority of the surveyed doctors were using some of tests which were known to them as unnecessary [15]. The most frequent explanations given for this practice were that such tests were an aid to clinical judgment and were necessary for medicolegal reasons. However we don’t believe this is an excellent explanation to practice defensive medicine. In the literature many studies suggested that there is significant association between medical litigation and specialty, however and inconsistent with *Ortashi et al*. our study did not show any significant different in the practice of defensive medicine among different specialties [2]. Also in line with *Ortashi et al*. our study revealed no significant correlation between litigation and different investigated variables. Defensive medicine brings with it exponential increases in the costs associated with clinical practice. This is explained by poor communication and other causes of medical litigation such as poor decision making. Doctors’ decisions for their patients are strongly affected by concerns of possible legal consequences. Doctors therefore practice defensive medical decision making aiming to protect themselves from blame and litigation and some fears can be healthy and can lead to adaptive responses. Good process should help create trust, rapport and alliance by showing respect for the patient. Lack of ethical issues as well as hospitals guidelines will lead to increase in the medical litigation and thus defensive medicine; this is obviously observed among our respondents since only 45.3 % and 39.3 % reported having high risk and ethical committees respectively.

## Conclusion

The majority of Sudanese doctors who are working in Obstetrics and Gynaecology had the impression that litigation against doctors are increasing, almost one third had a direct experience of litigation and more than two thirds reported practicing one or another form of defensive medicine. There should be strategic plan to reduce the practice of defensive medicine and medical litigation against doctors.

## Additional file

Additional file 1:
**Concept of Defensive Medicine and Litigation among Sudanese Doctors Working in Obstetrics and Gynecology** (DOCX 28 kb)
