# Supplementary material for: Concept of defensive medicine and litigation among Sudanese doctors working in obstetrics and gynecology
Source: BMC Med Ethics. 2016 Feb 9;17:12. doi: 10.1186/s12910-016-0095-3 (PMC4748468; doi:10.1186/s12910-016-0095-3)
Supplement: Additional file 1: — Concept of Defensive Medicine and Litigation among Sudanese Doctors Working in Obstetrics and Gynecology (DOCX 28 kb) [file 12910_2016_95_MOESM1_ESM.docx]

##### Concept of Defensive Medicine and Litigation among Sudanese Doctors Working in Obstetrics and Gynecology

**Introduction**

**We are a group of researchers (Dr. Abdel Aziem AbdAllah, Dr. Muawia E. Hummeida, Prof. Gamal Khalid, Dr. Wissal Nabag sand others) concern about the public health and quality of care provided by health care personnel in Sudan. WELCOME AND THANKS YOU for response to participate in this study. This questionnaire is designed to assess the concept of defense medicine and medico-legal litigation among Sudanese doctors working in OBGYN. And by answering our questions; you will improve the quality of care. It will take about 20 minutes to be filled. Please note that your information IS NOT personally identifiable and all responses are CONFIDENTIAL.**

| **Interview Date: …………………………………………………………………………..** |
| --- |

| Section 1 : Personal data |
| --- |

| **Write answer here** | **Respondent Choices** | **Question** | No |
| --- | --- | --- | --- |
|  | ……………….….years | Age | 1 |
|  | ……………………years | Duration of work | 2 |
|  | 1.Consultant  2.Specialist  3.Registrar | Qualification | 3 |
|  | 1. Male 2. Female | Gender | 4 |
|  | 1. Yes 2. No | Insurance coverage | 5 |

| Section 2 : Information on place of work an Experience |
| --- |

|  | 1. Teaching hospital  2. Rural hospital | Place of work | 6 |
| --- | --- | --- | --- |
|  | 1. Yes 2. No | Guidelines/protocol | 7 |
|  | 1.Yes 2.No | Hospital High Risk Committee | 8 |
|  | 1. Yes 2- No | Hospital Ethical Committee | 9 |
|  | 1. Yes 2. NO | Auditing system (Morning meeting, Discharge clinic, ect) | 10 |
|  | 1. Yes 2. No | Are you working in area characterized by blame culture? | 11 |
|  | 1. always 2.Usually 3.Sometimes | Application of informed consent | 12 |
|  | 1. always 2.Usually 3.Sometimes | Application of high risk consent for high risk patients | 13 |
|  | 1. always 2.Usually 3.Sometimes | Documentation | 14 |

- *The high risk consent defined as the consent which is taken in case of serious / complicated / risky / new - surgeries or procedures; for removing any organ; in high risk patients; for proceeding with a surgery / procedure in spite of any abnormal parameters of the patient.*

| Section 3 : Defensive Medicine Concept |
| --- |

| **Write answer here** | **Respondent Choices** | **Question** | No |
| --- | --- | --- | --- |
|  | 1.yes  2. No | Do you know the concept of defensive medicine? | 15 |
|  | 1.yes  2. No | Did you prescribed unnecessary medication to avoid litigation or criticism? | 16 |
|  | 1.yes  2. No | Did you experience unnecessary refer to avoid litigation or criticism? | 17 |
|  | 1.yes  2. No | Did your refuse to manage high risk patient because of your fear from litigation? | 18 |
|  | 1.yes  2. No | Did you ask unnecessary investigation to avoid criticism | 19 |
|  | 1.yes  2. No | Did you experience unnecessary surgical procedure to avoid criticism or litigation | 20 |
|  | 1.yes  2. No | Did you avoid high risk surgical procedure because of fear of criticism or litigation | 21 |

| Section 4 : Litigation in OBGYN |
| --- |

| **Write answer here** | **Respondent Choices** | **Question** | No |
| --- | --- | --- | --- |
|  | 1.yes  2. No  3. I don’t know | Is the litigation in OBGYN increasing? | 22 |
|  | 1.yes  2. No | Have you direct experience of litigation? | 23 |
|  | 1.Fetal distress  2. Shoulder dystocia  3. Maternal death  4. Perinatal death  5. Misdiagnosis of cancer  6. Injury to the visera  7. other (specify)……………….. | If yes what was the source? | 24 |
